# Effects of Red Lentil Flour Gels on the Development and Rheological Parameters of Dough and Bread Texture

**DOI:** 10.3390/gels11110894

**Published:** 2025-11-08

**Authors:** Sorina Ropciuc, Cristina Ghinea, Ana Leahu

**Affiliations:** Faculty of Food Engineering, Stefan cel Mare University of Suceava, 720229 Suceava, Romania; sorina.ropciuc@fia.usv.ro (S.R.); analeahu@fia.usv.ro (A.L.)

**Keywords:** composite flours, lentil flour, lentil gels, healthier bread, rheology, texture analysis

## Abstract

This study aims to investigate the role of red lentil flour gel in the development of dough and bread texture. The flour was obtained from untreated (FLU), blanched (FLS), and fermented (FLF) red lentil seeds. Subsequently, wheat flour was replaced with lentil flour in different percentages (0, 2, 4, 6, 8, and 10%), and the α-amylase activity of the flour samples was determined. The rheological properties of the dough during the fermentation process (dough development and gas formation and retention, elastic (G′) and viscous (G″) moduli) were also investigated. The hardness, resilience, cohesiveness, and elasticity of the bread samples were obtained using a TVT-6700 texturometer (Perten Instruments, Hägersten, Sweden). The results showed that α-amylase activity was stronger and the falling number decreased as the amount of lentil flour added increased (from 506 ± 2.50 s (control sample) to 386 ± 1.25 s for 10% FLU and to 403 ± 0.60 s for 10% FLF), except for the FLS samples (which ranged from 518 ± 2.92 to 559 ± 2.81 s). Lentils can disrupt the gluten network in dough, and it has been observed that dough quality was influenced by the addition and treatment of lentils: the maximum height of the dough decreased (from 53.8 mm (control sample) to less than 35 mm) as the percentage of wheat flour replaced by lentil flour increased. In contrast, the amount of gas formed was greater than in the control sample, demonstrating the positive effect of lentil flour on dough fermentation. Textural analysis showed positive effects at moderate concentrations of up to 6% lentil flour. Thus, bread hardness decreased from 1933 ± 0.13 (control sample) to 1849 ± 0.75 for 6% FLU and 1911 ± 0.56 for 6% FLF. The results showed that the use of 4% blanched or fermented lentil flour in dough gives it superior properties compared with regular dough, which leads to improved properties in baked goods.

## 1. Introduction

Lentils (*Lens culinaris*), native to Mesopotamia (Turkey), are cultivated worldwide [1], with over 7 million tons of dried lentils produced in 2023 [2]. In 2023, India, with over 1.5 million tons, and Turkey, with 474,000 tons of dry lentils, were the main producers in Asia, while Canada, with over 1.6 million tons, was the main producer in America. However, in the same year, the world’s largest producer of dry lentils was Australia, with over 1.8 million tons, according to the FAO [2]. Lentils are an environmentally sustainable crop, being drought-resistant and capable of growing in various soil types with a pH between 5.5 and 9 [3]. Pulses (chickpeas, lentils, dry peas, and beans) are consumed for their high protein, fiber, and mineral content [4]. Affrifah et al. [5] reported that dry lentils contain 63.35% carbohydrates (with 35–53% starch [6]), 24.63% protein, 10.70% dietary fiber, and 1.06% total lipids, while Gharibzahedi et al. [7] determined 25.90% protein, 59.01% carbohydrate, and 2.7% fats in red lentils and 27.30% protein, 61.5% carbohydrate, and 2.5% fats in green lentils. Lentils are rich in protein, contain most of the essential amino acids [8], and are also a source of β-glucans (soluble dietary fibers), which lower the glycemic index and maintain body weight [9]. According to the FAO [10], the highest pulses supply quantity was recorded in Africa (6.75 kg/capita) and Asia (4.76 kg/capita) in 2022. The lowest level of pulses consumption worldwide was reported in Europe (14.9 g/day) [11]. The consumption of pulses has health benefits and is associated with lower cancer-related mortality and reduced risks of cardiovascular and coronary diseases, and some authors have noted that lentils, in particular, may reduce the risk of type II diabetes [11]. However, they contain trypsin and chymotrypsin inhibitors, tannins, saponins, lectins, phytic acid, and oxalic acid, which can lead to a number of antinutritional problems, such as gastrointestinal discomfort, stomach upset, poor nutrient absorption, negative effects on protein bioavailability and digestibility, and reduced mineral bioavailability [12]. Also, lentils may contain allergens that can have adverse effects on human health from mild skin irritation to severe anaphylaxis [8]. Lentils can be red, green, yellow, or Spanish brown, and are relatively quick to prepare [13]. Heat treatment (cooking) and fermentation can significantly reduce the levels of antinutrients [12,14]. According to Aghababaei et al. [8], fermentation improves the digestibility of legume proteins. In addition to direct consumption through cooking, lentils can be processed into flour and protein, starch, and fiber.

Lentil flour enhances food by retaining water and fat and increases the percentage of protein and dietary fiber in the food product [13]. This flour is rich in starch (amylose and amylopectin) and protein, thus greatly influencing the rheological properties of dough [15]. According to Ahmed [15], during lentil flour processing, the starch dispersions undergo gelatinization and form a gel. The texture of food products is also affected due to the gel-forming properties of the starch and proteins from the lentil flour. Most legumes are rich in protein (i.e., >20%) and soluble fiber, but the consumption of legumes is hindered by long cooking and preparation times. To increase the consumption of alternative flours like those from fruits, vegetables, and legumes, studies propose adding them to common foods like baked goods, which can boost their nutritional value, such as protein and fiber [16]. The novelty of lentil flour gel lies in its ability to form a structured three-dimensional matrix by combining lentil proteins and starch, offering different functionalities compared with flours, isolates, or starch used separately [8,17]. In food products lentil flour is used as a thickener, binder, gelling agent, and/or stabilizer [6]. Leguminous flour such as lentil flour can be used (usually less than 10%) in bread making [18]. Previous research on fortifying wheat flour for baking with legume flour indicates that legume seed (chickpea, field bean, lentil, and pea) flours can interact with wheat flour dough in various ways [19,20,21].

Bread quality is influenced by starch gelatinization [22], which is a process that involves the simultaneous application of heat and time to break down starch crystallites [15]. Adding chickpea, lentil, and bean flour can increase water absorption due to the higher water retention capacity of legume flour, influencing gelation and binding [23]. Kotsiou et al. [18] replaced wheat flour with 10, 15, and 20% flour from sprouted and roasted lentils and evaluated the properties of the dough and fortified bread using rheometry, calorimetry, FTIR spectroscopy, and texture analysis. The partial gelatinization of lentil flour can occur when bread is made from a mixture of 80% wheat flour and 20% roasted–sprouted lentil flour. The addition of lentil flour increased water absorption and dough development time and decreased dough stability, according to Kotsiou et al. [18]. Marchini et al. [24] observed that at higher levels of wheat flour replacement, a general worsening effect on dough rheology may occur. Lentil flour has a significant influence on the rheological properties of doughs and the textural parameters of finished products. The aim of this paper was to investigate the effect of red lentil flour (obtained from untreated lentil seeds, blanched lentil seeds, and fermented lentil seeds) on the development and rheological parameters of dough and bread texture.

## 2. Results and Discussion

### 2.1. Characterization of Mixtures in Terms of Falling Number

In the study of the α-amylase activity of flour samples, the moisture content of the mixtures was determined by thermobalance. Thus, the falling number was established: 9.9% for wheat flour and untreated lentil flour (FLU), 11.4% for wheat flour and blanched lentil flour (FLS), and 11.8% for wheat flour and fermented lentil flour (FLF). Table 1 presents the falling number of flour mixture samples with different proportions of lentil flour.

Sivakumar et al. [25] stated that a wheat flour falling number between 300 and 450 s or higher is considered acceptable for bread production, while a falling number below 200 s is only suitable for breadcrumbs. A higher falling number value indicates lower amylase activity and vice versa. The activity of α-amylase can greatly reduce the quality of flour [26]. However, He et al. [27] stated that a small amount of α-amylase does not have a negative impact on baking quality. α-amylase activity is high when the falling number is below 150 s, moderate when it is between 200 and 250 s, and low when it is above 300 s [28,29]. Bread obtained from flours with low α-amylase activity is undeveloped and has a low volume and a dry crumb, and the flour must be corrected by adding enzymes [28]. The high activity of α-amylase can produce thin gels [26]. Mixtures of untreated lentil flour (FLU) have on average a lower falling number than the control sample and the other types of mixtures, which determines that the α-amylase activity is stronger. The falling number was between 386 and 394 s; this number decreased with the increasing percentage of lentils added. The results are close to those obtained by Korovyansky et al. [30]: 380 s for 5% lentil flour and 387 s for 10% lentil flour, with a decrease to 335 s for 25% lentil flour. The lentil flour was hydrated prior to testing (FLS, FLF), so that when determining the falling number, this flour came into contact with water again and created a three-dimensional structure through gelation, which was denser than in FLU, disrupting the determination of the enzymatic activity potential. Heat treatments influence gelling ability and according to Ma et al. [31], legume flours form weak gels at 10% concentration. Fermentation may adversely affect the gelation activity of lentil flours, as these flours already contain partially or fully gelatinized starch, according to Badia-Olmos et al. [32]. The FLU and FLF samples obtained better results than the control sample (Table 1), which highlights the influence of enzymes in lentil flour on the reduction in starch into directly reducing sugars. The obtained results showed that the concentration of lentil flour does not significantly influence its efficiency on the falling number. This is because the enzymes in lentil flour act beneficially to reduce this index, and their higher concentration should directly reduce this index, but as the concentration of lentil flour increases, the gluten-free proteins in the lentil flour also undergo hydration, which in turn creates a gel that is stronger than starch and thus increases the falling number.

### 2.2. Characterization of Fermentation Power of Doughs with Different Concentrations of Lentil Flour

The dough fermentation properties measured with a rheofermentometer are shown in Table 2. The H_m_ values of the FLU, FLS, and FLF samples were significantly lower compared with the control. Lentils are gluten-free and high in fiber, which can disrupt the gluten network of the dough, resulting in a less risen dough [33]. CO_2_ is produced during fermentation, and a lower H_m_ indicates that the gas was not sufficient or properly distributed in the air bubbles [33]. This means that the dough is not rising properly. The highest values for maximum dough height (H_m_) were obtained for the FLU samples (untreated lentil) compared with the other lentil flour samples (FLS, FLF), which means that the lentil treatment influences the dough quality. H_m_ decreased with an increasing percentage of wheat flour replacement by lentil flour. According to Sun et al. [34] the decrease in dough height could be due to decreased gas production, excessive hardness that prevented dough expansion, and reduced gluten matrix development that minimized the retention of CO_2_ formed during fermentation.

In the FLF samples, significant dough development is observed, but the percentage fall is very high, which indicates a poorer dough stability. Compared with the control sample, it can be observed that the percentage of drop is much lower in the lentil flour samples (FLU, FLS). This demonstrates the effectiveness of lentil flour in dough composition, specifically the effect on dough stability and gluten network. In the case of gas formation and retention, FLU samples (FLU–4, FLU–2) record the highest values, followed by FLF (FLF–2), and the lowest are observed for FLS (FLS–4, FLS–8, FLS–10). However, the FLU samples have lower gas retention values than the other samples, as can be observed by the retention coefficient (R), with the highest values being in the sample that formed the least FLS gases. Compared with the control sample, there is a marked difference in the amount of gas formed. Most of the samples have an amount of gas formed that is higher than the control sample, demonstrating the positive effect of lentil flour on dough fermentation. Interactions between fiber structure and wheat proteins influence the rheological behavior of doughs, and the addition of non-wheat raw materials reduces the dough’s retention capacity [23]. It has been pointed out that the more gas produced, the more CO_2_ is lost. Fermented red lentils have higher gas formation because fermentation breaks down complex carbohydrates and oligosaccharides into simpler, more easily fermentable sugars. They have lower gas retention because this process also alters the protein structure, weakening the dough’s gluten network, which makes it less capable of holding gas. In contrast, untreated seeds contain more indigestible compounds, and blanched seeds have modified proteins that can strengthen the dough’s structure, leading to less gas production but better retention. Flour obtained from vegetables such as lentils added to bread dough disrupts the starch–gluten matrix and leads to a reduction in the specific volume of the bread and an increase in the hardness and chewiness of the crumb. The rheological behavior of the dough is also influenced by the presence of fiber; the use of lentil flour can increase the proportion of fibers that interact with wheat proteins. In the case of doughs with added lentil flour, fermentation retained a total of 78.8% (FLU), 85. 1% (FLS), and 82.7% (FLF) of the total CO_2_ produced, values similar to those reported for vegetables (78.1%) by Bojňanská et al. [23]. The weakest flours in terms of their ability to retain the highest possible volume of gas in the dough were the samples with untreated lentil flour added.

### 2.3. Characterization of Dough Rheological Properties

**Figure 1:**
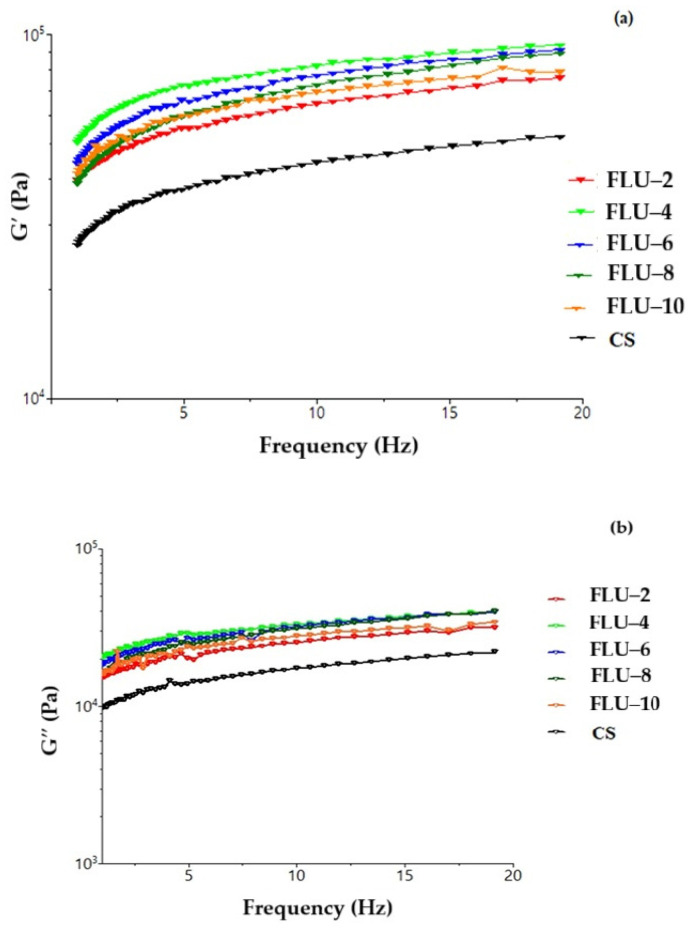
Variation in modulus of (**a**) elasticity (G′) and modulus of (**b**) viscosity (G″) with the angular frequency for dough samples with wheat flour (CS) and flour from untreated red lentil seeds (FLU) in different percentages.

The highest values were obtained from the lentil flour mixture of 4%, which means that this mixture is the most efficient for obtaining a dough with superior viscoelastic characteristics compared with the others (Figure 1). The 10% lentil flour mixture loses some of its viscoelastic properties, the reason being that the non-gluten proteins of lentils are overhydrated and have a weak link with the three-dimensional gluten structure. The 2% flour mixture having G′ and G″ indexes close to the control sample signifies a too low concentration of lentil flour and the inefficiency of its properties (Figure 1). Lentil proteins can form weak bonds or even strengthen the gluten structure, while insoluble fibers act as fillers in the network, contributing to increased dough rigidity [35]. As the proportion of lentils increases (up to FLU–10), the network becomes increasingly compact, which is reflected in the higher G values observed in the graph.

This effect can be interpreted positively from the perspective of the structural stability of the dough during technological processes (kneading, proofing, baking). However, an excessive increase in stiffness can negatively affect the extensibility of the dough and the volume of the final product [36]. Thus, the use of lentil flour in moderate proportions (e.g., FLU–4 or FLU–6) may represent an optimal compromise between improving the nutritional value and maintaining favorable technological properties.

**Figure 2:**
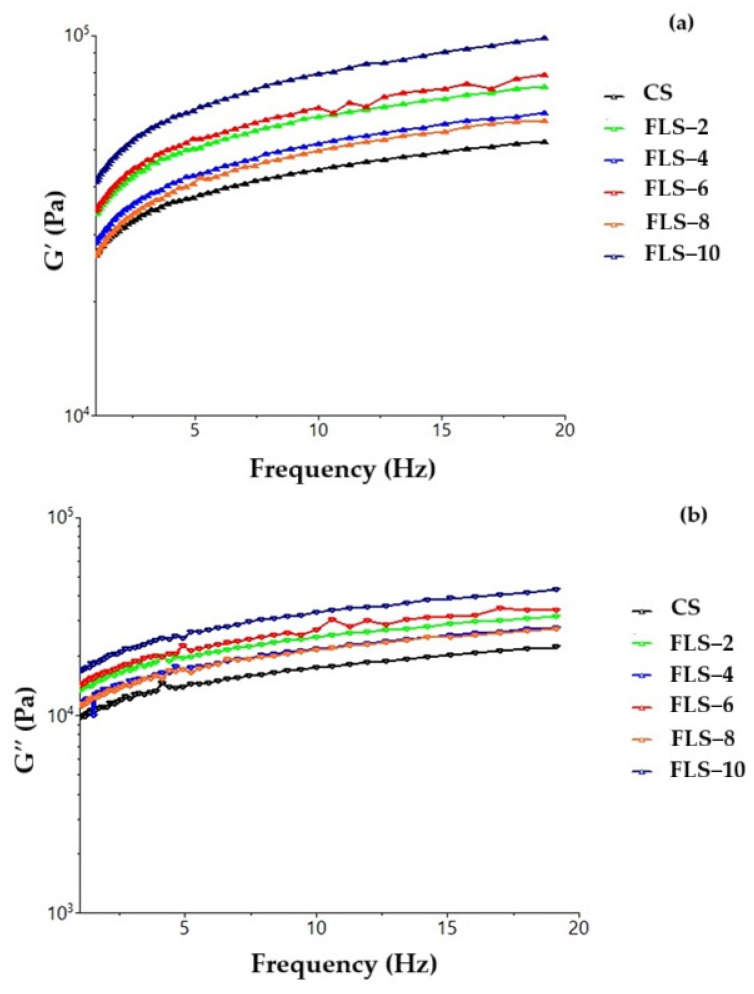
Variation in modulus of (**a**) elasticity (G′) and modulus of (**b**) viscosity (G″) with the angular frequency for dough samples with wheat flour (CS) and flour from blanched red lentil seeds (FLS) in different percentages.

The blanching of red lentil seeds was carried out to activate enzymes, but at the same time an undesirable hydration process of these seeds occurred, which influenced the rheological parameters of the dough. As in the case of the untreated lentils, the highest efficiency for the viscoelastic parameters of the dough is obtained with the lentil flour mixture with a concentration of 4%, with G′ and G″ indices much higher than the other samples (Figure 2). All values are higher than the control sample, which means that also blanching lentils provides beneficial rheological properties to the dough, due to the activated enzymes in lentils, which reduce hydrated starch to sugars, decrease dough density, and give better elasticity and viscosity. Figure 2a shows a progressive increase in G′ values with higher levels of lentil flour, indicating a strengthening of the protein matrix. This increase may be attributed to protein–protein interactions between lentil proteins and wheat gluten, as well as the contribution of insoluble fibers acting as structural fillers within the dough matrix [35,37]. A denser network contributes to enhanced elasticity, resulting in a firmer dough that can better maintain its structure during processing steps such as mixing, fermentation, and baking. Similarly, Figure 2b shows an increasing trend in G″ values with the addition of lentil flour, suggesting reduced viscous flow and greater resistance to deformation. These changes reflect an overall reinforcement of the dough’s viscoelastic properties, which may be beneficial for structural stability during technological processes [38].

**Figure 3:**
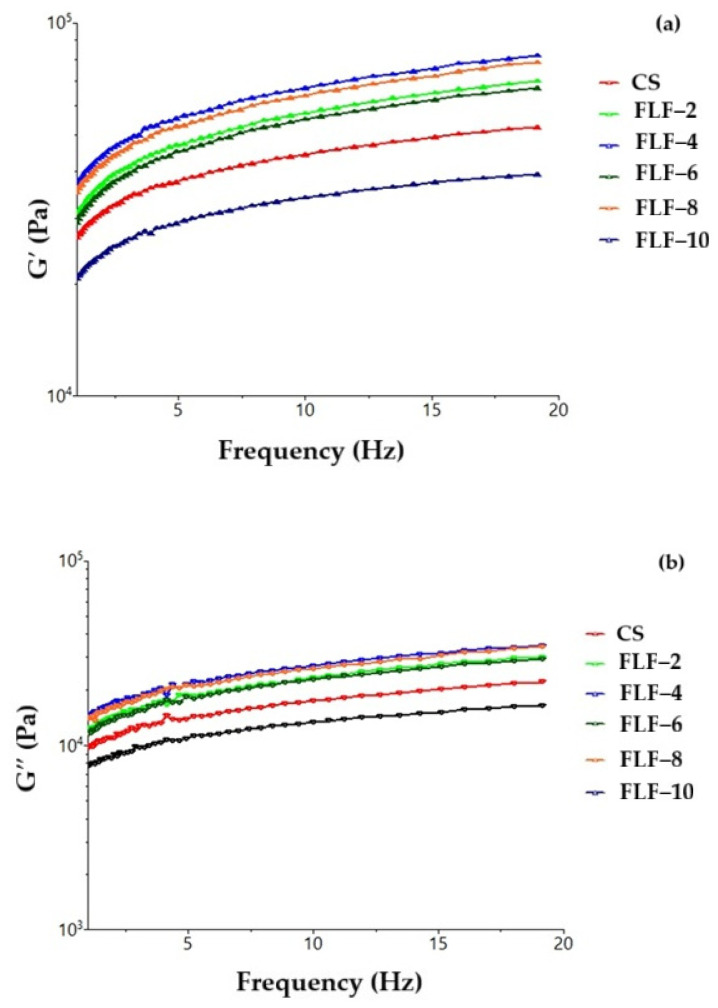
Variation in modulus of (**a**) elasticity (G′) and modulus of (**b**) viscosity (G″) with the angular frequency for dough samples with wheat flour (CS) and flour from fermented red lentil seeds (FLF) in different percentages.

From Figure 3, it can be seen that the greatest influence on the viscoelastic properties is from the flour with a 4% concentration of fermented red lentil seed. All curves follow an upward trend with frequency, a behavior typical for viscoelastic materials, where the internal structure becomes more rigid at high frequencies. Doughs with FLF are consistently more rigid than the control (CS), a sign that FLF contributes to the strengthening and stabilization of the dough’s internal network. The 10% lentil flour mixture has lower index values than the control sample, with the reason being overhydration during the applied treatment and the high concentration of non-gluten proteins; adding these effects together resulted in lower rheological properties than the control sample without lentil flour. After all the analyses carried out to study the behavior of the elastic and viscous modules, in all the samples, with minor exceptions, a more superior rheological behavior was evidenced compared with the control sample, which indicates the efficiency of using lentil flour to obtain a technologically good dough. The most effective concentration was found to be 4% lentil flour (Figure 3). The disadvantage of hydration during pre-treatments of blanched and fermented lentil seeds was observed, indicating lower modulus index values. Also, the effect on the enzymatic efficiency of the dough was noticed in all types of mixtures, which demonstrated the positive effect of using lentil flour in the formation of rheologically well-developed dough [39].

### 2.4. Textural Characterization of Baked Products from Red Lentil Flour Mixtures

The textural parameters of bread like hardness, resilience, cohesiveness, and elasticity were determined (Table 3). The hardness of the control sample was 1933 ± 0.13. The FLU samples had lower hardness values (between 1799 ± 0.57 for FLU–2 and 1915 ± 0.39 for FLU–10) compared with the control sample, as well as the FLS samples (except for FLS–10 with a hardness of 2076 ± 0.06), but also the FLF samples (except for FLF–10 with a hardness of 2212 ± 0.24). The samples that showed lower hardness values than the control sample highlight the positive effect of the use of lentil flour in the dough on the hardness of the bread. It was also observed that the hardness of the samples increased with an increasing percentage of lentil flour after investigating the lentil bread samples. The results showed that for bread elasticity, the positive effect of using lentil flour in the dough is largely highlighted, due to the higher values obtained in the samples with lentil flour compared with the control sample without the addition of lentil flour (988 ± 0.37). The highest value was obtained for FLF–2 (1171 ± 0.29) and the lowest for FLF–10 (895 ± 0.36), suggesting that in order to obtain better elasticity, the maximum limit will be a 6% concentration of lentil flour to wheat, since increasing the concentration of lentil flour already decreases the elasticity values. The influence of the addition of lentil flour to the dough on the textural parameters is considerable, with a positive effect in the case of moderate concentrations of up to 6% of lentil flour and a negative effect in the case of concentrations of more than 10% of lentil flour. Lentil flour increases the hardness of wheat bread, and the more flour is added, the greater the firmness due to the higher protein content that disrupts the gluten network, fibers, and resistant starch [20]. Lentil flour also causes thickening of the walls around air cells [40]. Starkute et al. [40] observed greater hardness in most lentil breads, except for samples containing 5% fermented ground lentils.

## 3. Conclusions

Lentil seeds have proteins, enzymes, and dietary fiber in their chemical composition, directly reducing carbohydrates, which has a significant positive influence on the rheological properties of doughs. Enzymes, acting on starch by reducing it, are beneficial for the formation of doughs with low alpha-amylase activity. This was demonstrated in the analysis of the FN index. Lentil flour in the doughs has the effect of creating a three-dimensional structure by interacting with the gluten, giving it superior properties compared with ordinary doughs. This was demonstrated in the analysis of the behavior of the viscous and elastic modulus using G′ and G″indicators. Lentil flour has a significant influence on dough during the development phase, acting on the enzymatic and microbiological processes. The amount of gas formed was significantly higher when lentil flour was used in doughs, and its development was beneficially influenced by the functional components of lentil flour. In the rheofermentometer analysis, it was observed that the dough formed with the addition of untreated lentil flour had the highest development and the lowest percentage of falling, and in the formation of gas, quantitatively it was the highest. All the samples showed positive effects compared with the control sample on the stability of the dough, expressed by the percentage of fall, and on the higher fermentation potential, expressed by the amount of gases produced during fermentation. In terms of dough development, the samples with a low lentil flour content (2% and 4%) (FLU–2, FLU–4, FLF-2, and FLS–2) were closest to the control sample, while for gas formation and especially gas retention, the use of 4 and 6% lentil flour would be more appropriate (FLS–4, FLS–6, and FLF–4). From a rheological point of view, samples with 4% lentil flour, regardless of whether they are treated or not, perform best. The addition of lentil flour reduces the hardness of bread and increases its elasticity for almost all samples, except for FLF, to which up to 6% lentil flour can be added. Lentil flour is a good way to improve baked goods, from a nutritional, functional, and sensory point of view.

## 4. Materials and Methods

### 4.1. Sample Formulation

In this study, untreated flour type 650 without improvers, enzymes, or other technological additives was used for the wheat–lentils flour blend. The protein content of the flour declared by the manufacturer is 11%. This type of flour was chosen to emphasize the properties of the legume powder in a classic dough.

Lentil flour was obtained by grinding lentil seeds which were treated in different ways. Red lentil seeds were used for the research and three types of lentil flour were obtained: untreated lentil seed flour (FLU), flour from blanched lentil seeds (FLS), and fermented lentil seed flour (FLF). Lentil seeds were blanched (FLS) in water at 90–100 °C for 5–10 min to deactivate peroxidases and lipoxygenases [41,42,43]. Then the seeds were dehydrated at 50 °C temperature using a Hendi Profi Line machine (Hendi, Brasov, Romania), at 550 W, for 24 h. After drying and cooling, the blanched lentil flour was again subjected to grinding. Lentil seeds were fermented (FLF) with lactic acid bacteria (*Lactobacillus delbrueckii ssp. bulgaricus* and *Streptococcus thermophilus*) for 24 h in water solution at a constant temperature of 28 °C [44,45]. The solution was then separated and the seeds were dried at room temperature for 24 h and subsequently ground. All flour samples were brought to the same grain size using s 500 μm mesh size sieve system. The wheat flour and lentil seed flour mixture samples are presented in Table 4.

**Figure 4:**
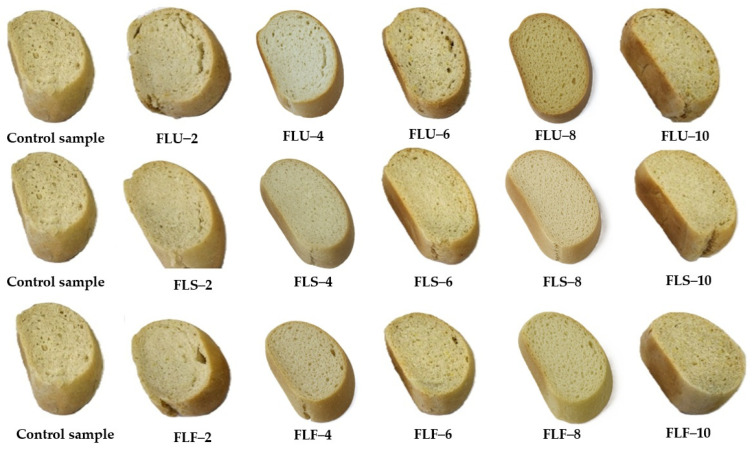
Sectional appearance of bread samples with red lentil flour mixtures (FLU—wheat flour and untreated lentil seed flour samples; FLS—wheat flour and flour from blanched lentil seeds samples; FLF—wheat flour and fermented lentil seed flour samples).

The sectional appearance of the bread samples with red lentil flour mixtures is illustrated in Figure 4.

### 4.2. Bread-Making

The bread recipe included flour mixture (80 g), water (40 g), sunflower oil (10 g), salt (1 g), and yeast (0.56 g). The technological process for all samples was carried out simultaneously and identically, with more precise details as follows: dosing of ingredients; kneading the dough—2 min; oil addition; kneading the dough—4 min; shaping the final shape and placing on trays; rest at a temperature of 28 °C for 80 min; baking in a convection oven, at a temperature of 180 °C for 20 min; and cooling at 20 °C for 120 min.

### 4.3. Determination of α-Amylase Activity of Flours

The method is based on rapidly gelatinizing a suspension of whole wheat flour or bran in a boiling water bath and measuring its liquefaction by α-amylase by determining the time required for a viscometric stirrer to fall a given distance. The higher the activity of the α-amylase, the stronger the liquefaction of the resulting mixture and the shorter the fall time of the viscometric stirrer. This time is the dropping number or index and is expressed in seconds. A falling number device (Perten Instruments, Hägersten, Sweden) was used according to SR ISO 3093:2010 [46]. A 7 g sample (wheat flour) with 15% moisture was weighed to the nearest 0.05 g. For the other moisture content of the sample, the weighed amount is modified so that the dry matter to total water ratio (including water through the sample to be analyzed) is constant. The weighed sample was placed in a viscometer tube and 25 mL of water at 20 ± 5 °C was added using a pipette and the tube immediately closed with a rubber stopper. It was shaken vigorously by hand 20 times or more if necessary to obtain a homogeneous suspension. The stopper was removed and the stirrer was placed in the tube, cleaning the wall of the tube so as to entrain the flour or bran particles into the suspension. The viscometer tube was immersed with the stirrer in the boiling water bath by opening the tube holder. The time recorder was triggered when the viscometer tube reached the bottom of the water bath. Exactly 5 s after introduction into the bath, the stirring of the suspension in the tube begins. After 59 s the stirrer has been brought to the upper position (or is raised automatically), and after 60 s it is released and under its own weight falls into the heated flour or groats glue at a speed which depends on the degree of liquefaction of the glue. The drop index is expressed as the total time, in seconds, calculated from the moment the viscometer tube is immersed in the water bath until the viscometer stirrer drops a given distance into the heated flour glue [46].

### 4.4. Measurement of Gas Production and Dough Development

The Rheofermentometer F3 (Chopin Rheo, Villeneuve-La-Garenne Cedex, France) was used to determine dough development and gas production throughout the fermentation process. For this, 250 g of flour, 7 g of yeast pressed into a suspension, and 5 g of salt and water according to the hydration capacity were placed in a kneading trough thermostated to 28.5 °C (Farinograph kneading trough). The flour was mixed with pressed yeast and water according to the flour’s moisturizing capacity for 1 min in the Farinograph (Brabender GmbH and Co., Duisburg, Germany). Then the mixer was stopped and all the flour particles were cleaned with a spatula for uniform hydration. The mixer was restarted for 6 min, adding salt progressively at the beginning of this period. At the end of kneading, the dough was drawn in and 315 g of dough was retained. The 315 g of dough was placed in the aluminum bowl of the machine. The aluminum dish was placed in the Reofermentometer enclosure (in the heated block) and the displacement sensor was placed on top of it, sealing the dish with the socket and the fixing screws. The following indicators were determined [47]: maximum dough height (H_m_, mm); percentage fall after 3 h ($\frac{\left(H_{m}-h\right)}{H_{m}},\;\%$), from the dough development chart; maximum height of gaseous production (H′_m_, mm); total carbon dioxide volume production (V_CO2total_, mL); volume of CO_2_ lost (V_CO2lost_, mL); and gas retention coefficient (Rc, %), from the gas formation and retention chart.

### 4.5. Dough Rheological Properties During Fermentation Process

The rheological analysis of the dough was carried out using a HAAKE RheoWin Mars 40 dynamic rheometer (Thermo-HAAKE, Karlsruhe, Germany). All dough samples consisted of 10 g of sample flour of the previously specified wheat–lentils concentrations and 6 g of water. All measurements were carried out at a temperature of 20 °C, using a rotor/plate geometry with a rotor diameter of 40 mm and a plate spacing of 2 mm. Determinations of the behavior of the elastic (G′) and viscous (G″) moduli at variable frequencies in the range 1–20 Hz were performed [48]. The dough can be studied without affecting its structure by dynamic rheology methods with small oscillations. Methods based on small deformations applied in oscillatory mode are commonly used to study formation processes and rheological properties. The dynamic response of viscoelastic fluids to oscillatory stimuli is characterized by two basic indices—the elastic storage modulus G′ and the elastic loss modulus G″ [49,50].

### 4.6. Texture Analysis of Bread

Bread texture parameters, such as hardness (maximum force registered during the first compression cycle), elasticity (measured as the distance of the detected height of the second compression divided by the original compression), cohesiveness (the ratio between the positive area of the second cycle and the positive area of the first cycle) [47], and resilience (the beginning of a sample’s elasticity) [51], were determined using the TVT-6700 texturometer (Perten Instruments, Hägersten, Sweden). A 45 mm cylindrical probe, operating at a speed of 5.0 mm/s and a trigger force of 5 g, was used. Bread samples were cut into 50 mm high slices and compression was performed at a displacement equal to 75% of the sample height at a pressing speed of 4 mm/s [48]. The test parameters were as follows [50]: 10.0 mm/s pre-test velocity, 5.0 mm/s test velocity, 5.0 mm/s post-test velocity, and 40% deflection.

### 4.7. Statistical Analysis

The number of replicates was as follows: three replicates for the falling number of flour mixture samples and bread texture parameters; single determination for characterizing the fermentation power of doughs with different concentrations of lentil flour and the characterization of the rheological properties of the dough. For the sample’s differentiation, the analysis of variance ANOVA (α = 0.05) was conducted using Minitab version 17 (State College, PA, USA).

## Figures and Tables

**Table 1:** Falling number of flour mixture samples with different proportions of lentil flour.

Lentil Seed Flour Percentage in the Flour Mixture	Wheat Flour Type 650	FLU ^1^	FLS ^1^	FLF ^1^
	Falling Number (s)			
0%	506 ± 2.50			
2%		394 ± 0.70 ^a^	559 ± 2.81 ^a^	501 ± 2.47 ^a^
4%		393 ± 0.75 ^a^	549 ± 1.91 ^b^	455 ± 1.61 ^b^
6%		393 ± 1.56 ^a^	534 ± 2.01 ^c^	433 ± 1.27 ^c^
8%		387 ± 1.20 ^b^	531 ± 2.30 ^c^	425 ± 0.50 ^d^
10%		386 ± 1.25 ^b^	518 ± 2.92 ^d^	403 ± 0.60 ^e^

^1^ FLU—wheat flour and untreated lentil seed flour samples; FLS—wheat flour and flour from blanched lentil seeds samples; FLF—wheat flour and fermented lentil seed flour samples. Different lowercase letters across columns denote a notable difference in average values (*p* < 0.05), as determined by one-way ANOVA analysis.

**Table 2:** Rheofermentometer indicators of dough development and gas formation and retention.

Samples	Dough Development *		Gas Formation and Retention *			
	H_m_ (mm)	(H_m_-h)/ H_m_ (%)	H’_m_ (mm)	V_CO2 total_ (mL)	V_CO2 lost_ (mL)	R (%)
Control sample	53.8	14.3	52.7	1171	127	89.2
FLU–2	50.6	1	65.2	1606	349	78.3
FLU–4	45.9	9.7	70.3	1676	421	74.9
FLU–6	39.1	2.8	60.5	1459	287	80.4
FLU–8	37.5	4.8	58.9	1420	279	80.4
FLU–10	35.9	3.5	57.3	1381	271	80.4
FLS–2	40	5.8	63.8	1501	297	80.2
FLS–4	38.1	7	54.1	1086	91	91.6
FLS–6	33.3	5.4	55.9	1245	179	85.6
FLS–8	28.5	4.6	47.8	960	145	84.9
FLS–10	26.6	4.3	44.7	896	150	83.3
FLF–2	42.8	21.5	75.2	1589	221	86.1
FLF–4	39.9	20.1	70.1	1481	239	83.8
FLF–6	38.9	18.3	63.6	1399	254	81.9
FLF–8	38.9	19.6	68.3	1443	269	81.3
FLF–10	35	16.6	66.2	1378	268	80.6

H_m_—maximum dough height; (H_m_-h)/H_m_—percentage fall after 3 h; H′_m_—maximum height of gaseous production; V_CO2total_—total carbon dioxide volume production; V_CO2lost_—volume of the CO_2_ lost; Rc—gas retention coefficient. * Single determination.

**Table 3:** Textural parameters of bread with different percentages of treated red lentils.

Samples	Hardness (g)	Resilience	Cohesiveness	Elasticity
Control sample	1933 ± 0.13 ^c^	1.00 ± 0.01 ^ef^	0.51 ± 0.13 ^a^	988 ± 0.37 ^n^
FLU–2	1799 ± 0.57 ^o^	1.18 ± 0.08 ^bcd^	0.58 ± 0.05 ^a^	1114 ± 0.12 ^g^
FLU–4	1815 ± 0.66 ^m^	1.20 ± 0.04 ^bcd^	0.59 ± 0.07 ^a^	1117 ± 0.17 ^f^
FLU–6	1849 ± 0.75 ^k^	1.29 ± 0.05 ^bc^	0.60 ± 0.09 ^a^	1111 ± 0.55 ^h^
FLU–8	1871 ± 0.22 ^i^	1.35 ± 0.02 ^ab^	0.58 ± 0.02 ^a^	1051 ± 0.24 ^k^
FLU–10	1915 ± 0.39 ^e^	1.50 ± 0.06 ^a^	0.58 ± 0.03 ^a^	1060 ± 0.35 ^j^
FLS–2	1809 ± 0.36 ^n^	1.06 ± 0.07 ^def^	0.56 ± 0.03 ^a^	1142 ± 0.99 ^c^
FLS–4	1841 ± 0.50 ^l^	1.15 ± 0.04 ^cde^	0.58 ± 0.03 ^a^	1037 ± 0.1 ^l^
FLS–6	1857 ± 0.05 ^j^	1.26 ± 0.08 ^bc^	0.59 ± 0.04 ^a^	1135 ± 0.13 ^e^
FLS–8	1911 ± 0.25 ^f^	1.30 ± 0.06 ^bc^	0.57 ± 0.02 ^a^	1103 ± 0.05 ^i^
FLS–10	2076 ± 0.06 ^b^	1.35 ± 0.08 ^ab^	0.55 ± 0.06 ^a^	1019 ± 0.09 ^m^
FLF–2	1877 ± 0.52 ^h^	0.94 ± 0.06 ^f^	0.48 ± 0.07 ^a^	1171 ± 0.29 ^a^
FLF–4	1894 ± 0.11 ^g^	1.15 ± 0.08 ^cde^	0.55 ± 0.04 ^a^	1148 ± 0.30 ^b^
FLF–6	1911 ± 0.56 ^f^	1.24 ± 0.07 ^bc^	0.60 ± 0.06 ^a^	1140 ± 0.62 ^d^
FLF–8	1926 ± 0.23 ^d^	1.25 ± 0.03 ^bc^	0.58 ± 0.02 ^a^	903 ± 0.40 ^o^
FLF–10	2212 ± 0.24 ^a^	1.26 ± 0.01 ^bc^	0.53 ± 0.05 ^a^	895 ± 0.36 ^p^

Different lowercase letters across columns denote a notable difference in average values (*p* < 0.05), as determined by one-way ANOVA analysis.

**Table 4:** Wheat flour and lentil seed flour samples.

**Ingredients**	**Wheat Flour and Untreated Lentil Seed Flour (FLU) Samples**				
	FLU–2	FLU–4	FLU–6	FLU–8	FLU–10
Wheat flour	98%	96%	94%	92%	90%
Untreated lentil seed flour	2%	4%	6%	8%	10%
	**Wheat flour and flour from blanched lentil seeds (FLS) samples**				
	FLS–2	FLS–4	FLS–6	FLS–8	FLS–10
Wheat flour	98%	96%	94%	92%	90%
Flour from blanched lentil seeds	2%	4%	6%	8%	10%
	**Wheat flour and fermented lentil seed flour (FLF) samples**				
	FLF–2	FLF–4	FLF–6	FLF–8	FLF–10
Wheat flour	98%	96%	94%	92%	90%
Fermented lentil seed flour	2%	4%	6%	8%	10%

## Data Availability

The original contributions presented in the study are included in the article, further inquiries can be directed to the corresponding author.
